# Optimal Localization of Smart Aggregate Sensor for Concrete Damage Monitoring in PSC Anchorage Zone

**DOI:** 10.3390/s21196337

**Published:** 2021-09-22

**Authors:** Quang-Quang Pham, Ngoc-Loi Dang, Quoc-Bao Ta, Jeong-Tae Kim

**Affiliations:** 1 Department of Ocean Engineering, Pukyong National University, Busan 48513, Korea; bkdn06x3a@gmail.com (Q.-Q.P.); loi.ngocdang@gmail.com (N.-L.D.); qb.tabao@gmail.com (Q.-B.T.); 2 Urban Infrastructure Faculty, Mien Tay Construction University, 20B Pho Co Dieu Street, Vinh Long 890000, Vietnam

**Keywords:** smart aggregate, optimal sensor location, impedance-based damage monitoring, sensitivity analysis, prestressed concrete anchorage

## Abstract

This study investigates the feasibility of smart aggregate (SA) sensors and their optimal locations for impedance-based damage monitoring in prestressed concrete (PSC) anchorage zones. Firstly, numerical stress analyses are performed on the PSC anchorage zone to determine the location of potential damage that is induced by prestressing forces. Secondly, a simplified impedance model is briefly described for the SA sensor in the anchorage. Thirdly, numerical impedance analyses are performed to explore the sensitivities of a few SA sensors in the anchorage zone under the variation of prestressing forces and under the occurrence of artificial damage events. Finally, a real-scale PSC anchorage zone is experimentally examined to evaluate the optimal localization of the SA sensor for concrete damage detection. Impedance responses measured under a series of prestressing forces are statistically quantified to estimate the performance of damage monitoring via the SA sensor in the PSC anchorage.

## 1. Introduction

The prestressed concrete (PSC) structure has a higher loading capacity, thinner body, and more crack-resistance performance than the traditional reinforced concrete structure. These prominent characteristics are generated via the support of internal force that is induced by the prestressing force, which is transferred into the concrete domain by the anchorage zone [1,2,3]. The compression force is applied to immature concrete during the prestressing stage, thus leading to damage in the anchorage zone [2,3]. The concrete damage induced by high tensile bursting stress is often initiated at the internal area due to the dispersion of prestressing force into the concrete domain [2,3]. The internal concrete damage leads to structural degradation (e.g., reinforcement corrosion, strand corrosion, and prestressing loss), deteriorating concrete members. 

The incipient concrete damage in the anchorage zone should be detected in a timely fashion in order to adjust the construction process and to assure the integrity of the PSC structure. Sensing and monitoring the location of the damage should then be predetermined before embedding sensors into the anchorage zone. The optimal sensor placement could enhance the accuracy and reliability of the monitored result [4,5,6]. It also helps to optimize the number of sensors installed in the target structure. 

In past decades, damage detection in PSC structures has been the concern of many researchers and practitioners [7,8,9]. Strain-based methods have been commonly used to directly estimate prestress force levels based on well-defined strain-stress relationships in steel strands [10,11]. Among strain sensors, fiber optic sensors using Fiber Bragg grating (FBG) have high durability and sensitivity, and they show promise for use in prestressing monitoring [11,12]. However, due to difficulties in encapsulation of FBG into steel-strands (so-called smart strands), the widespread application of smart strands has been hindered. Among local damage detection methods, impedance-based approaches have emerged as a promising way forward [13,14]. To date, impedance monitoring has been extensively implemented for damage detection in concrete structures (e.g., concrete crack, prestress loss, rebar corrosion, and concrete strength degradation) [15,16,17,18,19,20,21]. The method uses a coupling interaction between a PZT (lead zirconate titanate) sensor and a target structure in order to acquire electromechanical (EM) impedance responses, which represent local structural characteristics of the inspected region [22,23]. As a fundamental component, any change to the structural condition leads to change in the impedance responses. Thus, local damage can be identified by quantifying variation in the impedance responses measured before and after damage.

Many researchers have attempted to monitor impedance response-induced damage from PZT sensors placed on the surface of concrete structures [16,24,25]. The surface-mounted sensors could detect impedance signatures induced by local damage near the surface; however, they were not sensitive enough to monitor variation in the impedance responses that were induced by incipient damage in the anchorage zone [26,27]. Moreover, the impedance responses obtained from the surface-bonded PZTs were inevitably affected by ambient conditions, which required a complicated compensation process [28,29]. Recently, many research efforts have been made in terms of embeddable piezoelectric sensors for structural health monitoring, such as concrete hydration monitoring [21,30,31,32] and impact damage detection [33,34,35]. To ensure the integrity of PZT sensors, fragile PZT patches were protected by layers of epoxy or asphalt lacquer. The sensor-layers devices were then installed in small concrete blocks before being embedded in the target structures.

Installed in the host structure, the embedded-PZT sensor could become an ideal approach for monitoring impedance features induced by incipient internal damage in the concrete matrix. Some perspectives show the feasibility of the embedded-PZT sensor for health monitoring of the PSC anchorage zone. Firstly, the embedded-PZT sensor directly receives impedance responses induced by the change in internal stress fields and the occurrence of inner damage events [15]. Secondly, PZT patches are coated by protection layers (i.e., epoxy and concrete block) to ensure the integrity of the sensors and to reduce the effect of noisy ambient conditions [36,37,38]. Thirdly, the embedded-PZT sensors are less subjected to variation in temperature than the surface-bonded PZT sensors, as a result of the protection of the concrete layer. 

The applicability of the embedded-PZT sensors has not been investigated for impedance-based monitoring of incipient inner cracks in the PSC anchorage zones. Optimal sensor localization should be examined for the PSC anchorage zone in order to enhance the accuracy of the monitoring results. This study aims to investigate the feasibility of smart aggregate (SA) sensors and their optimal locations for impedance-based damage monitoring in prestressed concrete (PSC) anchorage zones. Firstly, numerical stress analyses are performed on the PSC anchorage zone to determine the location of potential damage induced by prestressing forces. Secondly, a simplified impedance model is briefly described for the SA sensor in the anchorage. Thirdly, numerical impedance analyses are performed to explore the sensitivities of a few SA sensors in the anchorage zone under the variation of prestressing forces and under the occurrence of artificial damage events. Finally, the practicality of the SA sensor is experimentally investigated on a real-scale PSC anchorage zone by evaluating the optimal localization of the SA sensor for impedance-based concrete damage detection. Impedance responses measured under a series of prestressing forces are statistically quantified to estimate the performance of damage monitoring via the SA sensor in the PSC anchorage.

## 2. Concrete Anchorage Zone under Prestress Force

### 2.1. FE Model of Concrete Anchorage Zone

**Figure 1:**
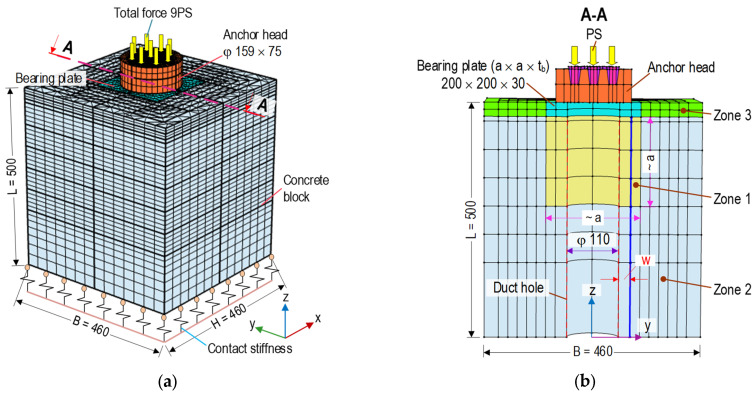
FE model of anchorage zone (dimension in mm): (**a**) Geometry and meshing, (**b**) Plane A-A.

As shown in Figure 1, a finite element (FE) model of a 9-strand concentric concrete anchorage zone was simulated to obtain stress responses under prestress forces. The anchorage zone was a combination of an anchor head (159 mm in diameter and 75 mm in height) with nine conical wedges, a bearing plate (a × a × t_b_ = 200 mm × 200 mm × 30 mm), and a concrete block (width × height × length = B × H × L = 460 mm × 460 mm × 500 mm). A cylindrical hole (φ 110 mm) was placed at the center of the concrete block as the duct carrying prestressed steel strands (see Figure 1b). Each strand had a nominal diameter of φ 15.24 mm and a tensile strength of 1860 MPa [39]. The anchorage zone was designed to withstand the prestress force 9 PS = 1170 kN (PS = 130 kN per strand). The material properties of the anchorage zone parts were listed in Table 1.

As shown in Figure 1a, the anchorage zone components were modeled by three-dimensional elements. The complete mesh of the FE model consisted of 42,724 elements which included 3208 elements for the anchor head, 888 elements for the bearing plate, and 38,628 elements for the concrete domain. As the boundary condition, the bottom surface of the FE model was modeled by assigning the contact stiffness with *k_x_ = k_y_* = 2.5 × 10^15^ (N/m/m^2^) and *k_z_* = 5.0 × 10^15^ (N/m/m^2^) [40]. A linear elastic model was commonly used to perform the stress analysis in the prestressed anchorage zone [2,3]. In this analysis, the materials of the multi-strand anchorage (i.e., bearing plate and anchor head) and the concrete block were assigned as homogeneous and linear elastic behaviors for the determination of stress distributions in the anchorage zone under prestressing force.

As shown in Figure 1b, the PSC anchorage was examined for three sub-zones (Zones 1–3) to analyze stresses induced by the prestress force [1,2,3,41]. Zone 1 (a local zone with a size of a mm × a mm × a mm), ahead of the bearing plate, was subjected to high compressive stress and tensile hoop stress. The failure in this zone would be formed as a crushing pattern caused by insufficient compressive strength of concrete. Zone 2, away from the anchorage device, was subjected to lateral tensile bursting stress due to the dispersion of the prestress force. A tensile concrete crack would be formed along the tendon path in this region. Zone 3, beside the bearing plate, was subjected to tensile spalling stress that occurred along the edges of the PSC anchorage. This study focused on the internal concrete damage (i.e., cracking) induced by the tensile-bursting stresses. It was also assumed that Zone 1 was confined with reinforcement that was sufficient to prevent the concrete crushing.

### 2.2. Stress Distribution Induced by Prestress Force

**Figure 2:**
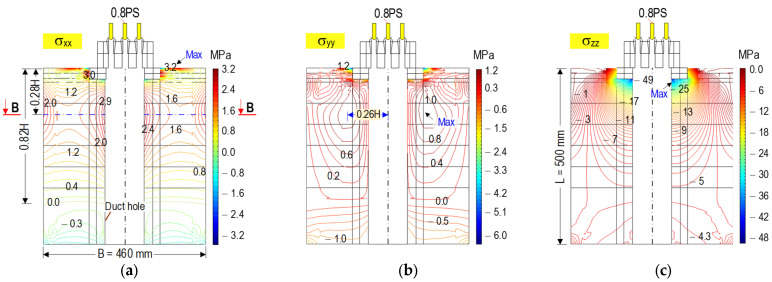
Stress contours in anchorage zone under prestress force 0.8 PS (plane A-A in Figure 1): (**a**) Tensile hoop stress; (**b**) Tensile bursting stress; (**c**) Axial compressive stress.

Figure 2 shows the contour of three stress components in the anchorage zone (tensile hoop stress σ_xx_, tensile bursting stress σ_yy_, and axial compressive stress σ_zz_) induced by the prestress force 0.8 PS. The maximum tensile hoop stress was observed at the top surface of the concrete anchorage (see Figure 2a). It was locally concentrated surrounding the duct hole. The significant stress values occurred at the distance of 0.28 H (plane B-B) away from the top concrete surface. The stress was reduced to zero at a distance of 0.82 H. Similar to the tensile hoop stress, the maximum bursting stress was localized at the cut plane B-B (see Figure 2b). The highest stress value was determined at a distance of 0.26 H away from the central axis along the tendon path of the anchorage. The zero-stress plane was the same as the tensile hoop stress. The axial compressive stress achieved the maximum value at the bottom contact surface between the bearing plate-concrete block, and it rapidly decreased as it spread out into the structure (see Figure 2c).

**Figure 3:**
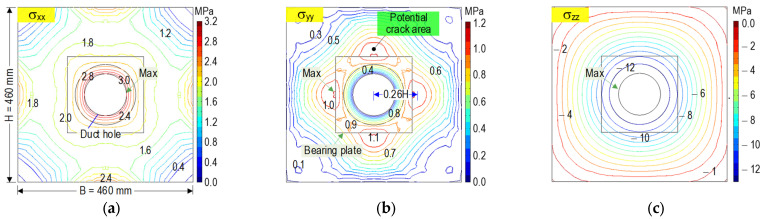
Stress contours on B-B plane under prestressing force 0.8 PS (x-y section): (**a**) Tensile hoop stress; (**b**) Tensile bursting stress; (**c**) Axial compressive stress.

Figure 3 shows the stress contours analyzed at plane B-B (see Figure 2a). The tensile hoop stress had a maximum value around the duct hole which reduced toward the corners of the anchorage (see Figure 3a). The bursting stresses were concentrated close to the interface between the local zone (Zone 1) and the general zone (Zone 2). The tensile bursting stresses were propagated toward the outer surfaces of the anchorage zone (see Figure 3b). Internal cracks would occur at the anchorage zone as the tensile bursting stress exceeds the concrete tensile strength [2,3]. Therefore, the areas of high bursting stresses were potential for the occurrence of internal cracks in the anchorage zone (see Figure 3b). As shown in Figure 3c, the compressive stresses got the highest value around the duct hole and gradually decreased over the whole plane.

**Figure 4:**
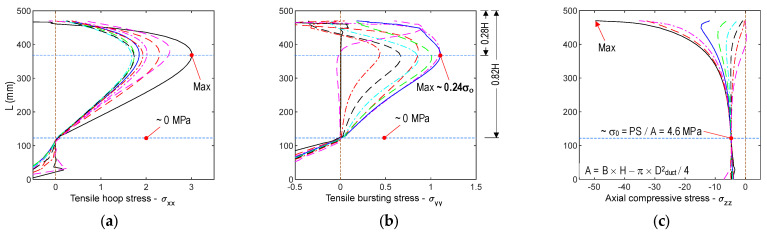
Stress distribution of concrete anchorage zone along tendon path (unit MPa): (**a**) Tensile hoop stress; (**b**) Tensile bursting stress; (**c**) Axial compressive stress.

Figure 4 shows the stress distributions along the tendon path in the anchorage zone. As shown in Figure 4a,b, the maximum values of the tensile hoop stress and the tensile bursting stress coincided with the stress contours shown in Figure 2 and Figure 3. The maximum tensile bursting stress was about 0.24 σ_o_, where σ_o_ = 9 PS/A denotes the uniformly distributed stress at the bottom surface of the anchorage with the cross-section A (see Figure 3c). This value was close to the results from the previous studies [2,3]. As shown in Figure 4c, the axial compressive stress was rapidly reduced along the tendon path, and it achieved the constant value σ_o_ at a distance away from the top surface of 0.82 H.

## 3. Smart Aggregate for Impedance Monitoring in Anchorage Zone

Based on the analyzed results in Section 2, the internal tensile damage (i.e., inner crack) could occur in the anchorage zone during the stressing procedure. Therefore, monitoring the internal tensile damage during the stressing process is essential to ensure the integrity and safety of the anchorage zone. A three-dimensional (3D) finite element (FE) model was simulated for the PZT-embedded smart aggregate (SA) sensors, and subjected to the variation of prestressing forces and the occurrence of tensile damage in the PSC anchorage. 

### 3.1. Impedance Model for PZT-Embedded SA Sensor

**Figure 5:**
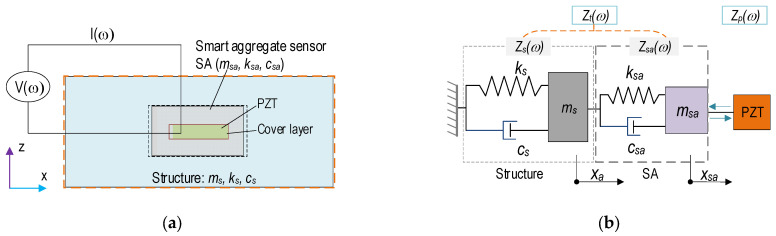
PZT-embedded SA sensor for impedance monitoring: (**a**) SA-embedded structure; (**b**) 2-DOFs impedance model.

Figure 5 shows the concept of impedance monitoring in a PSC structure via the PZT-embedded SA sensor [42,43]. A covered PZT is embedded into the center of a small concrete block before concreting. The SA sensor is placed at a potential damage location in a concrete structure. It then acquires impedance responses from the interaction between the PZT-SA and the monitored structure (see Figure 5a). The interaction can be simplified as a 2-DOFs impedance model, as illustrated in Figure 5b. In the impedance model, the terms *x_sa_* and *x_s_* represent motions of the SA and the structure, respectively. Note that the thin-protected layer of PZT is ignored. Structural parameters are denoted as *m* (mass), *k* (spring stiffness), and *c* (damping coefficient). The subscripts *sa* and *s* denote the smart aggregate and the concrete structure.

The coupling structural-mechanical (SM) impedance, *Z_t_* of the SA and the monitored structure can be simplified [26]:

(1) $$Z_{t}\left(\omega \right)=\frac{K_{11}\left(\omega \right)K_{22}\left(\omega \right)-K_{12}^{2}\left(\omega \right)}{i\omega K_{22}\left(\omega \right)}$$

where the terms *K_ij_* (*i,j* = 1–2) contain structural parameters of the SA sensor and the structure [23], as follows: *K_11_ =* −*ω^2^m_sa_ + iωc_sa_ + k_sa_*, *K_12_ =* −*iωc_sa_* − *k_sa_*_,_ and *K_22_ =* −*ω^2^m_s_ + iω(c_sa_ + c_s_) + (k_sa_ + k_s_).* The electromechanical impedance is a function of the structural mechanical (SM) impedance of the SA-structure and that of the PZT sensor, *Z_p_* [22]:

(2) $$Z(\omega )=\frac{V\left(\omega \right)}{I\left(\omega \right)}={\left\{i\omega A_{p}\left[{\hat{\varepsilon }}_{33}^{T}-\frac{1}{Z_{p}(\omega )/Z_{s}\left(\omega \right)+1}d_{31}^{2}{\hat{Y}}_{11}^{E}\right]\right\}}^{-1}$$

where *A_p_* is the geometric parameters of piezoelectric patch; ${\hat{\varepsilon }}_{33}^{T}$ is the complex dielectric constant of at zero stress; *d_31_* is the piezoelectric constant in 1-direction at zero stress; ${\hat{Y}}_{11}^{E}$ denotes the complex Young’s modulus of PZT patch at the zero electric fields, and *ω* is in the sweeping frequency range. As noted in Equation (2), the impedance responses *Z(ω)* contains the SM impedance (*Z_p_*(*ω*)) of the PZT patch and the SA-structure (*Z_t_*(*ω*)). Once the electric and mechanical properties of the PZT patch keep constant, any external effects (e.g., concrete damage) would exert an effect on its impedance responses, thus enabling the applicability of the PZT-embedded SA for monitoring damage detection in the concrete structure.

### 3.2. FE Model of SA-Embedded Concrete Anchorage Zone

#### 3.2.1. Anchorage Zone with SA Sensor

**Figure 6:**
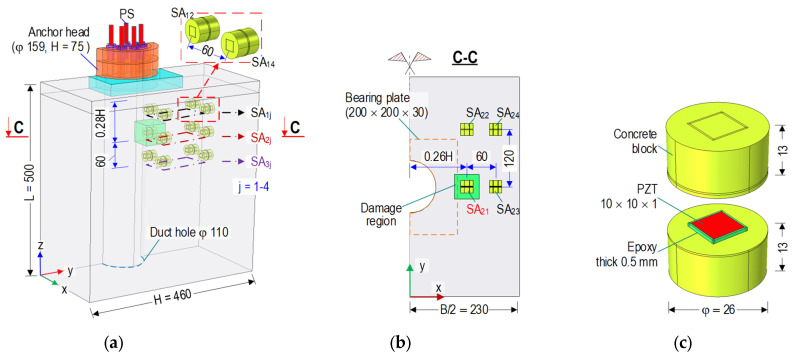
SA sensors and tensile damage region in anchorage zone (dimension in mm): (**a**) ½ FE model of anchorage; (**b**) Cut plane C-C; (**c**) Detail of SA sensor.

As shown in Figure 6a, the anchorage zone in Section 2 was utilized to analyze the sensitivity of the SA sensors under the variation of prestressing forces and the occurrence of inner artificial tensile damage. Considering the geometrical properties and the applied forces, only half of the 3D FE model of the anchorage zone was analyzed using Comsol Multiphysics. As shown in Figure 6c, a SA sensor consisted of a PZT patch (size 10 mm × 10 mm × 1 mm) coated by an epoxy layer (thickness 0.5 mm) and a small concrete block (diameter φ 26 mm × height 26 mm) made up of cement-sand mortar. The coated PZT was positioned in the middle of the SA sensor. Twelve laterally-placed SA sensors were embedded into the anchorage zone (see Figure 6a). To ensure the integrity of the SA sensor (concrete block), the strength of the concrete block should be larger than that of the host structure [44]. Material properties of the concrete block were selected as follows: *E* = 28.6 GPa (Young’s modulus), *v* = 0.2 (Poisson’s ratio), and *ρ* = 2400 kg/m^3^ (mass density). For the analysis of impedance responses, the simulated materials (concrete block, SA sensor, and anchorage system) were assumed as elastic homogeneous mediums [16]. 

The SA sensors were modeled to be located at the three layers along the tendon path. In addition, each layer had four sensors. As indicated in Figure 6a, the symbol SA_ij_ denotes the SA sensor of the i^th^ layer and the j^th^ position. For example, SA_11_ was located in Layer 1 at the first position. The sensors SA_2j_ in Layer 2 coincided with the plane B-B (0.28 H from the top surface of the anchorage), containing potential tensile damaged areas, as described previously in Figure 2 and Figure 3. The SA_1j_ in Layer 1 and the SA_3j_ in Layer 3 placed 60 mm away (upward and downward) from the SA_2j_. Figure 6b shows in detail the cut plane C-C of the SA_2j_ sensors and a damage region placed in Layer 2. 

As analyzed in the previous section, the locations of damage coincided with the SA_21_ sensor and its vicinity in Layer 2 (see Figure 6a,b) at the prestressing force 1.2 PS. The artificial damage zone (a size of 52 mm × 52 mm × 52 mm) was assumed at about twice the volume of SA. The location of the damage zone was simulated at the medium that had the highest tensile bursting stress (see Figure 2b and Figure 3b). It is noted that the damage could occur during construction processes (e.g., material faults or overloading). Since the material properties of SA sensors (i.e., concrete block) were higher than those of the concrete anchorage, the region in which damage occurs surrounds the SA sensor. The FE model consisted of 110,583 elements, including 108 elements for the 9-wedges, 664 elements for the anchor head, 400 elements for the bearing plate, 90,644 elements for the concrete block, 3967 elements for the damaged locations, and 14,800 elements for the twelve SA sensors. The quadratic hexahedron elements were used for the coated PZT (i.e., PZT patch and epoxy layer) of the SAs and the 9-wedges. In addition, the quadratic tetrahedron elements were used for the remaining domains of the FE model. The material properties of concrete and steel were selected, as described in the previous section. In terms of the PZT patch for impedance analysis, the material properties of PZT 5A were listed in Table 2. The contact stiffness was assigned for the bottom surface of the FE model, as described in the previous section.

#### 3.2.2. Simulation Scenarios for SA Sensor Localization

As listed in Table 3, two cases (Cases 1–2) were simulated to investigate the sensitivity of the PZT-embedded SA sensors in the anchorage zone. Case 1 was selected to estimate the sensitivity of the SA sensors based on stress distributions in the anchorage zone under the prestress force 0.8 PS for each of the nine strands. Impedance signatures were obtained from the three SA sensors: SA_11_, SA_21_, and SA_31_ (i.e., one sensor for each layer). Case 2 was selected to estimate the sensitivity of the SA sensors based on tensile concrete damage. Artificial damage was simulated near the SA_21_ by reducing the structural stiffness of the damaged medium to 1% [45]. The prestress force was applied as 1.2 PS. The impedance signals were numerically analyzed for all twelve SA sensors: SA_1j_, SA_2j_, and SA_3j_ in Layers 1–3 (see Figure 6a). The impedance signals were acquired in the frequency range of 100–600 kHz by applying a harmonic voltage 1V on the top surface of the PZT sensor. In the searched range, the frequency range of 150–300 kHz shows two clear resonant impedance peaks. This range was selected to analyze the impedance features of SA sensors.

### 3.3. Optimal Location of SA Sensors for Damage Monitoring

#### 3.3.1. Impedance Responses of SA Sensors under Prestress Forces

Resonant frequency ranges of impedance responses contained meaningful structural information [16,27]. In this study, the frequency range of 150–300 kHz was selected to perform impedance analyses. There were two resonant frequency peaks, Peaks 1–2, corresponding to frequency values around 200 kHz and 250 kHz, respectively. This range was also used to quantify the change in impedance signatures induced by the prestressing forces. 

**Figure 7:**
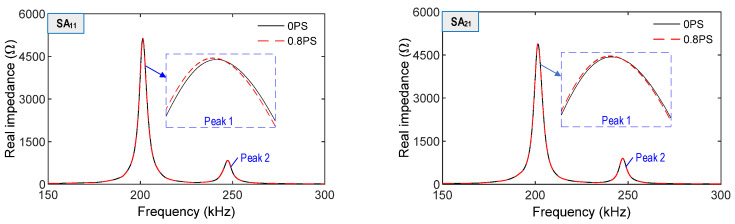

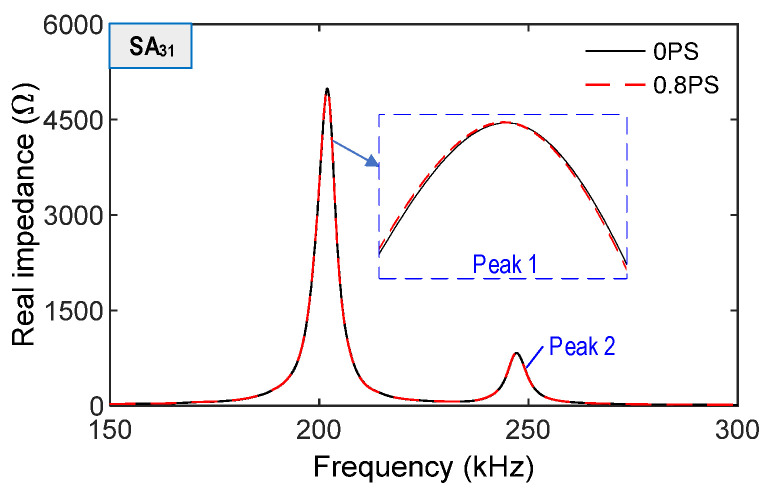
Numerical impedance responses of SA sensors under prestress forces.

In Case 1, impedance signals of SA_11_, SA_21_, and SA_31_ were acquired in the range of 150–300 kHz, as shown in Figure 7. The impedance signals were slightly varied under the variation in the prestress forces. The alteration of signals was relatively high at SA_11_ near the bearing plate.

**Figure 8:**
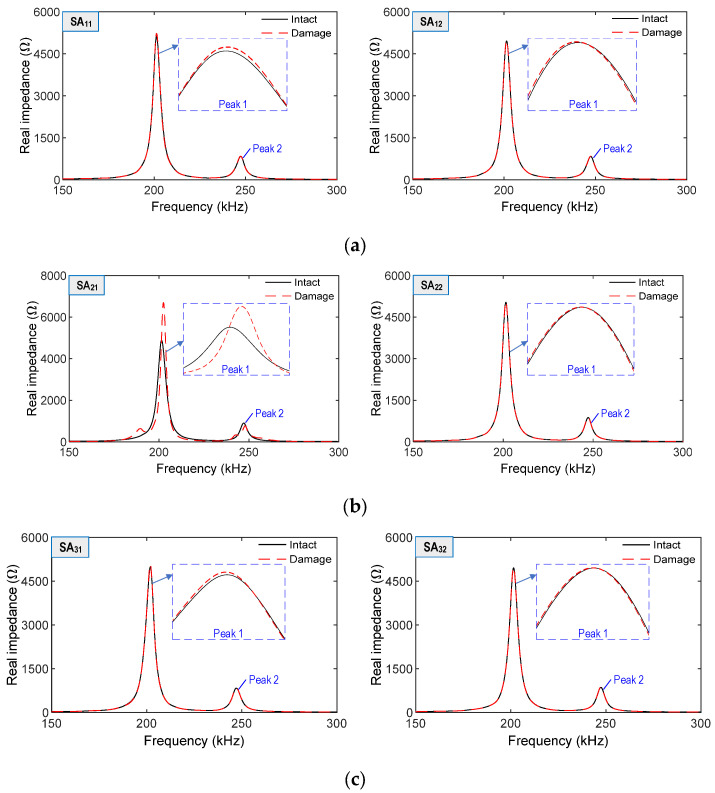
Numerical impedance responses of SA sensors due to local damage: (**a**) SA sensors on Layer 1; (**b**) SA sensors on Layer 2; (**c**) SA sensors on Layer 3.

In Case 2, impedance signatures of all twelve SA sensors were acquired in the range of 150–300 kHz, as shown in Figure 8. The impedance responses of SA_21_ (which covered by the concrete damage zone in Layer 2) were the most sensitive among the twelve SAs (see Figure 8b). Other SAs placed 60~100 mm away from the local damage had relatively small changes in terms of impedance responses. For example, the signals of SA_11_ (60 mm away) and SA_12_ (85 mm away) were little changed due to the damage (see Figure 8a).

#### 3.3.2. Optimal Location of SA Sensors in Anchorage Zone

Changes in impedance signatures (frequency shifts and varied impedance magnitude) were commonly quantified by using the RMSD (root-mean-square-deviation) damage metric to characterize structural damage [46]:

(3) $$RMSD\left(Z,Z^{*}\right)=\sqrt{\left(\sum_{k=1}^{N}{\left[Z^{*}(\omega_{k})-Z(\omega_{k})\right]}^{2}\right)/\sum_{k=1}^{N}{\left[Z(\omega_{k})\right]}^{2}}$$

where *Z(ω_k_)* and Z*(ω_k_) are impedance signatures recorded at the *k^th^* frequency before and after structural damage, respectively; and *N* is a number of the measured frequency points in the spectrum. Where the RMSD value is zero, this indicates that no damage exists, and, otherwise, the structure is damaged.

**Figure 9:**
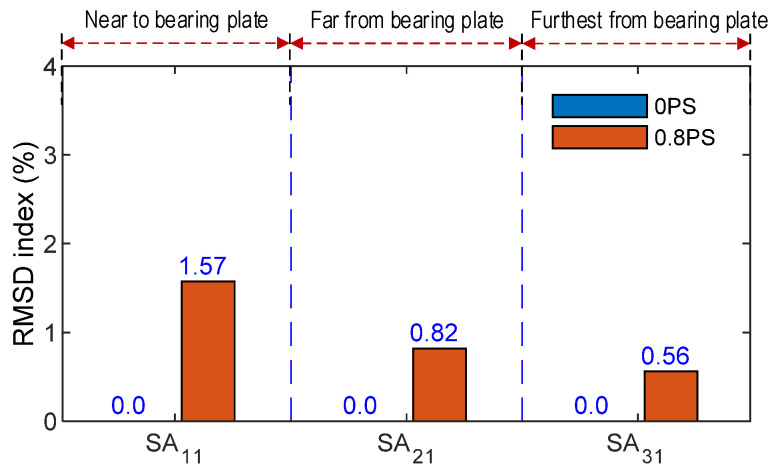
Impedance features of SA sensors along tendon path under prestress force.

RMSD indices were used to quantify the variation of impedance responses acquired from the SA sensors. In Cases 1–2, RMSD indices were calculated for the frequency range of 150–300 kHz. Figure 9 shows the RMSD indices computed from the impedance responses of the three sensors, SA_11_, SA_21_, and SA_31_, which were measured before and after the alteration of prestress forces (Case 1). The RMSD magnitude of SA_11_ was relatively higher than the two others. It confirmed that the SA placed close to the bearing plate experienced more stress variation than others. Knowing that more stress changes lead to more impedance variations [15,47], the SA sensors should be installed close to the bearing plate to sensitively monitor changes in the prestress forces.

**Figure 10:**
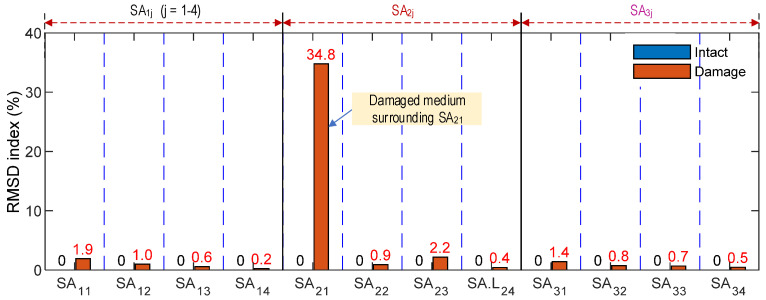
Impedance features of SA sensors in anchorage zone under damage.

Figure 10 shows the RMSD indices computed from the impedance changes of the twelve SA sensors, which were obtained for the simulation of Case 2. The highest RMSD magnitude was observed at the sensor SA_21_, which was covered by the concrete damage medium (see Figure 6a). Meanwhile, the RMSD magnitudes of other SA sensors were insensitively small under the damage event. The sensitivity of the impedance responses (obtained from the SA sensors at the vicinity of the concrete damage) could be significantly affected by the damage occurrence. The damage locations in the anchorage zone under the prestress forces could be accurately detected via the impedance responses of the SA sensors.

## 4. Experimental Evaluation of SA Sensor for Damage Monitoring in PSC Anchorage Zone

### 4.1. Prototype Design of SA Sensor

**Figure 11:**
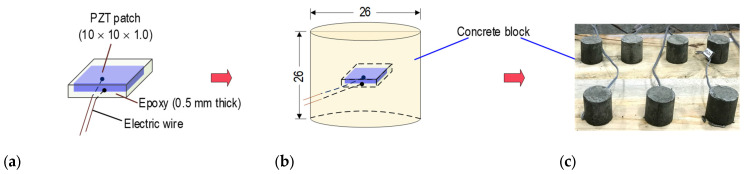
Fabrication process of PZT-embedded SA sensor for impedance monitoring (unit in mm): (**a**) Protected PZT patch; (**b**) SA sensor size; (**c**) Fabricated SA sensors.

To ensure the durability and sensing capability, the PZT sensor should be waterproof before being embedded into concrete structures. Figure 11 shows the prototype design of a PZT-embedded SA sensor for impedance measurement in PSC structures [42,48]. The PZT patch had the size of 1 cm × 1 cm × 0.1 cm, and it was protected by an epoxy layer of 0.5 mm thickness for waterproof and electric insulation (see Figure 11a). An electric wire was soldered on the top and bottom surfaces of the PZT patch. The covered PZT patch was then placed into a cylindrical mold to form the size of φ 2.6 cm × 2.6 cm (see Figure 11b). For the mixture of materials and concrete properties, as listed in Table 4, Mixture 1 was selected for the SA sensor. Figure 11c shows the sample of SA sensors at seven days after casting. 

### 4.2. Analysis of Optimal SA Sensor Location in PSC Anchorage Zone

#### 4.2.1. Design of SA-Embedded PSC Anchorage Zone 

**Figure 12:**
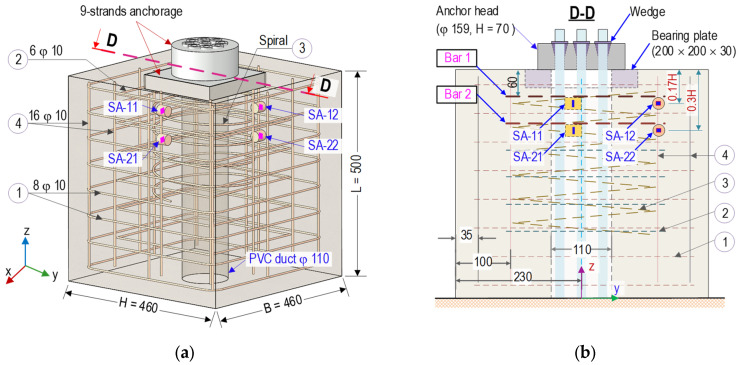
PSC anchorage configuration equipped with SA sensors (dimension in mm): (**a**) 3-D view; (**b**) Cut plane D-D.

A full-scale experiment was performed to evaluate the feasibility of the SA sensors for damage monitoring. As schematized in Figure 12a,b, a PSC anchorage zone was designed to resist compressive forces induced by anchoring nine pre-tensioned strands. The anchorage system included a 9-strand anchor head (φ15.9 cm × 7 cm) with conical wedges and a steel bearing plate (20 cm × 20 cm × 0.3 cm). The experimental anchorage model was designed with the geometric parameters, as shown in Figure 12a. Also, Figure 12b illustrated the detailed locations of SA sensors in the anchorage. The concrete block had B × H × L = 46 cm × 46 cm × 50 cm (width × height × length) designed with a cylindrical hole (φ 110 mm) at the center for passing multiple strands. The reinforcement was designed as follows: (1) eight orthogonal stirrups φ10 (*l* = 1760 mm and space @ = 60 mm), (2) six orthogonal stirrups φ10 (*l* = 1280 mm and @ = 60 mm), (3) a spiral φ10 (*l* = 4890 mm and @ = 50 mm), and (4) sixteen longitudinal rebars φ10 (*l* = 820 mm).

Mixture 2 (listed in Table 4) was selected to build the concrete anchorage block. It was designed with relatively low strength (σ_c_ = 16.7 MPa) to enable the simulation of concrete damage. Material properties of the concrete and reinforcement were listed in Table 5. In addition, the material properties of the anchorage components (i.e., anchor head, wedges, and bearing plate) were defined as: *ρ* = 7850 kg/m^3^ (mass density), *ν* = 0.33 (Poisson’s ratio), and *E* = 200 GPa (elastic modulus).

**Figure 13:**
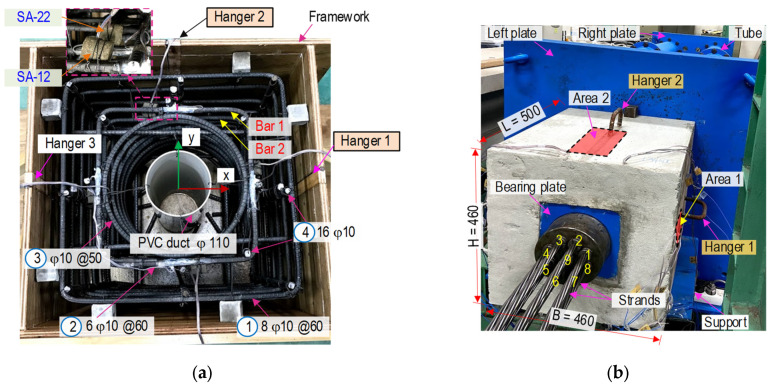
Setup of anchorage zone with SA sensors (dimension in mm): (**a**) Reinforcement and SA sensors installation; (**b**) Setup of anchorage zone on stressing frame.

Figure 13a shows the planning of the SA-embedded anchorage model. Figure 13b shows the anchorage zone installed on a full-scale test frame. The stressing frame was designed to bear the compressive force (about 3000 kN) induced by the prestressed anchorage. Details on the stressing frame were detailed in the previous study [27]. Nine prestressing strands (φ 15.2 mm, 7-wire strand, and Grade 270 low-relaxation steel) were installed in the frame (Strands 1–9, as seen in Figure 13b). As highlighted in the figures, two target areas (Areas 1–2) close to Hangers 1–2 were utilized to observed surface-crack development during prestressing strands. Material properties of the strands were outlined in Table 5 [39]. Each strand was installed with a load cell to measure the prestress force (applied by jacking systems) during the experiment. 

#### 4.2.2. Deployment of SA Sensors

As shown in Figure 12, the four SA sensors were installed in the PSC anchorage to detect internal concrete damage induced by the prestress forces. The SA sensors were placed at potential damage locations (analyzed in the previous section) caused by high tensile bursting stress. As shown in Figure 12b, two SA sensors were attached to the first inner stirrup layer (Bar 1). Two other sensors were affixed to the second layer (Bar 2). Bars 1 and 2 were distanced at approximately 60 mm and 120 mm to the top concrete surface, respectively. The four SA sensors were labeled SA-11 and SA-12 on Bar 1 and SA-21 and SA-22 on Bar 2. 

#### 4.2.3. Test Scenarios for Impedance Monitoring

**Figure 14:**
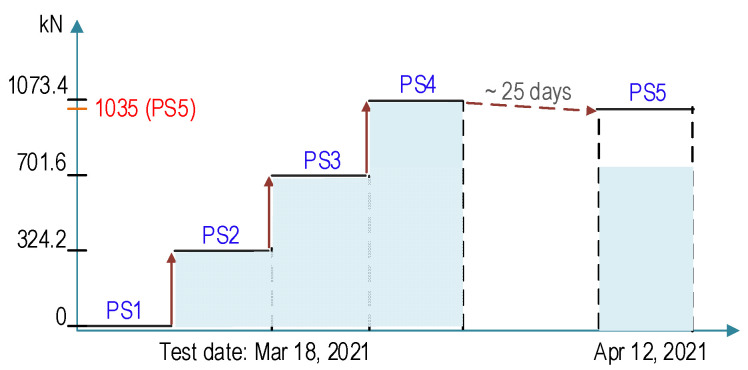
Prestress-force loading scenarios for impedance monitoring.

**Figure 15:**
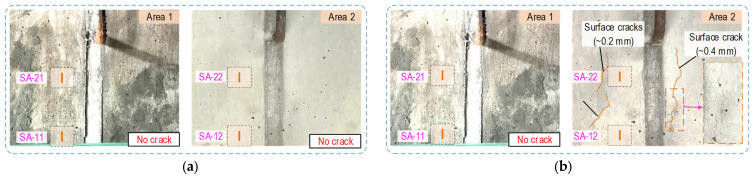
Surface cracks observed on anchorage surface under prestress-force cases: (**a**) Crack observation under PS4; (**b**) Crack observation under PS5.

As shown in Figure 14, the four test cases (PS1–PS4) were conducted on 18 March 2021 (about 500 days of concrete age) to measure the impedance signals. In PS1, all nine strands were stressed by about 1.0 kN force to set strands and the anchorage on the steel frame. By the calibration of the indicator, the force was considered to be the baseline state (i.e., PS1 = 0 kN). In PS2, the prestressing force of 40 kN was applied to each strand to simulate the second force level. Each strand was continuously tensioned up to 80 kN (PS3) and 120 kN (PS4). After PS4, the prestress forces had been kept for about 25 days. During the period, the total prestress forces lost about 40 kN into the final prestress level PS5; meanwhile, concrete surface cracks were disclosed on the anchorage zone (see Figure 15). For PS1–PS5, the impedance signals were measured from the four SA sensors.

For the experiment, a wired impedance analyzer, HIOKI 3532, was employed to excite a harmonic voltage 1 V and measure impedance responses from the SA sensors in the resonant frequency range of 100–600 kHz (501 points). In each level of the prestress forces, four ensembles of impedance signals were recorded to compute the control threshold UCL (upper control limit) and determine the standard deviation of measured data. During the test, laboratory temperature was controlled at about 21.5 °C to minimize the effect of temperature variation on impedance signals. 

Figure 15 shows the visual observation of cracks on the two side surfaces (Area 1 and Area 2) near the SA sensors (see Figure 13a,b). The monitored regions were concentrated on Area 1, covering two sensors SA-11 and SA-21. Area 2 enclosed two others (SA-12 and SA-22). The surface cracks were not observed until the loading case PS4 (about 120 kN per strand). At 25 days since the PS4 loading, surface cracks occurred in Area 2 along with the prestress-loss about 40 kN in all strands (see Figure 15). The crack lines were concentrated in the region covering the sensor SA-22. From this observation, it is inferred that internal cracks would be formed surrounding the SA-22 sensor domain due to the loading case PS4. It is also inferred that the internal cracks were propagated toward the concrete surface Area 2. The losses of prestressing force (measured by nine load cells) could be induced by crack formation.

### 4.3. Impedance Signatures of SA Sensors under Prestress-Force Variation

#### 4.3.1. Impedance Responses of SA Sensors in Intact Case

**Figure 16:**
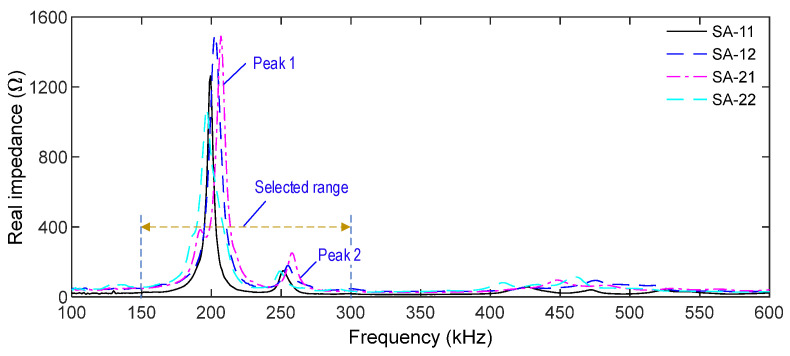
Impedance responses of SA sensors in frequency range 100–600 kHz under intact case.

As shown in Figure 16, the impedance responses of the intact cases were measured from the SA sensors in the frequency range of 100–600 kHz. For all SA sensors, high resonant impedance signatures were observed in the range of 150–300 kHz, which was consistent with the observation in the numerical impedance analysis in Section 3. The first and second resonant impedance peaks were around 200 kHz (Peak 1) and around 255 kHz (Peak 2). Noticing that the impedance spectra contain the information of local structural characteristics, the impedance frequency range (i.e., 150–300 kHz) was selected in order to monitor the PSC anchorage.

#### 4.3.2. Impedance Responses of SA Sensors under Loading Cases

**Figure 17:**
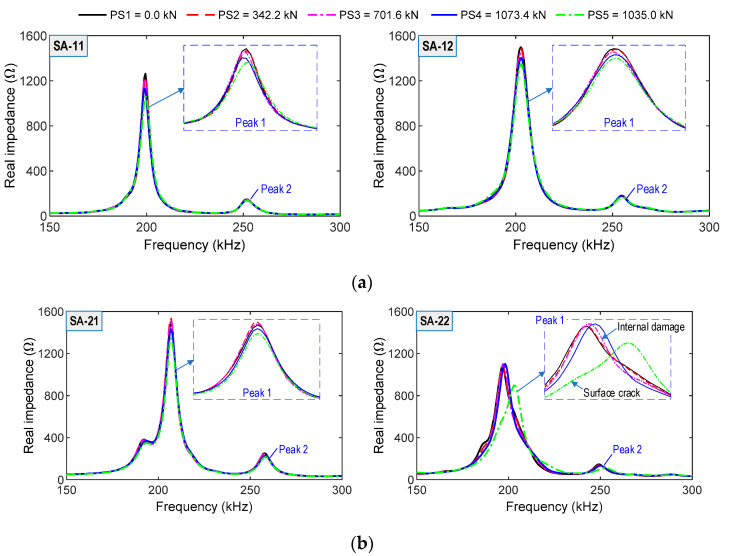
Impedance responses of SA sensors under loading cases PS1~PS5: (**a**) SA sensors attached on Bar 1; (**b**) SA sensors attached on Bar 2.

From the SA sensors, impedance signatures were measured in the range of 150–300 kHz under the prestress-force loadings PS1–PS5. Figure 17a,b shows the impedance spectra of the four SA sensors in the anchorage zone. The impedance signatures of SA-11 and SA-12 on Bar 1 (close to the bearing plate) and those of SA-21 on Bar 2 (below Bar 1 with a distance of 60 mm) were slightly shifted under the loading cases. Meanwhile, SA-22′s impedance signals (on Bar 2) were abruptly changed under the prestressing force PS3, thus indicating the local deformation of the concrete domain surrounding SA-22 (e.g., internal concrete damage [43,49]). The impedance responses were constantly varied under the applied force PS4 (internal damage evolution) and showed the most alteration under the test case PS5 (surface crack occurrence). The rushed variations in impedance signatures of SA-22 coincided with the disclosure of the surface cracks. Moreover, the observation was also consistent with the numerical impedance analysis, which impedance responses-induced inner concrete damage.

### 4.4. Damage Monitoring Using Impedance Features of SA Sensors

#### 4.4.1. Sensitivity of SA Sensors under Prestress-Force Variation

For the prestressing scenarios PS1–PS5, RMSD indices (see Equation (3)) were calculated in order to estimate changes in impedance signatures of the SA sensors. Four ensembles of the impedance signatures in the intact case (PS1) were employed to calculate the upper control limit (UCL) threshold. The UCL threshold was applied to reinforce the reliability of the experimental test [27], as follows:

(4) UCL=μ  +  3σ

where the mean *μ* and the standard deviation *σ* are computed from RMSD indices of the ensembles of impedance signals at the intact (PS1), in which 3*σ* represents damage detection with a 99.7% confidence level. Moreover, for each of the remaining test cases, PS2–PS5, four ensembles of impedance responses were utilized to determine the standard deviation. The RMSD indices were computed in the selected frequency range of 150–300 kHz for four SA sensors.

**Figure 18:**
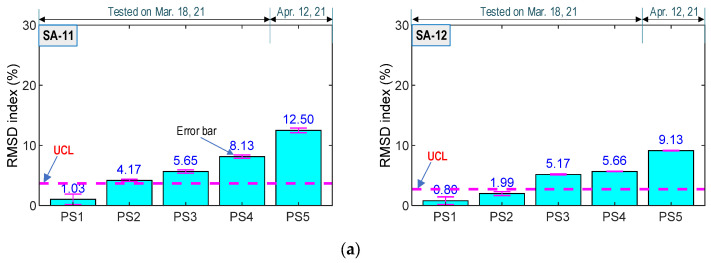

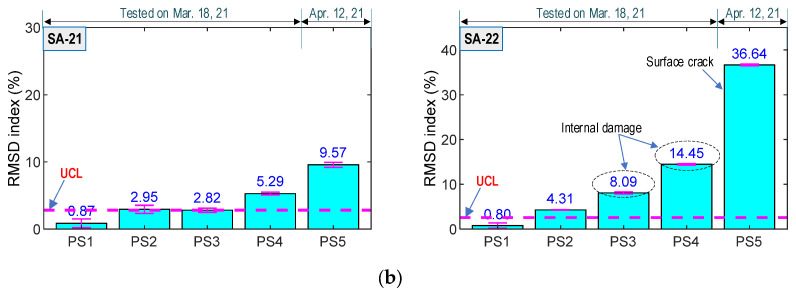
RMSD indices of impedance signals under loading cases PS1~PS5: (**a**) SA sensors attached on Bar 1; (**b**) SA sensors attached on Bar 2.

Figure 18 shows RMSD indices for the SA sensors in the anchorage under the force levels of PS1–PS5. The RMSD indices of the four sensors were not significant in the intact state (PS1); meanwhile, the RMSD magnitudes corresponding to the loading cases PS2–PS5 were beyond the UCL threshold. The error bars for the dispersion of measured data were relatively small, thus showing that the SA sensors could accurately monitor the variation of prestress forces and the occurrence of damage. 

The RMSD value of SA-11 on Bar 1 (see Figure 18a) was relatively higher than that of the SA-21 on Bar 2 (see Figure 18b). This observation was consistent with the numerical results in Section 3. Again, it can be confirmed that the concrete domain surrounding smart aggregates on Bar 1 (closer to the bearing plate) experienced more stress variation than those on Bar 2. Moreover, the RMSD magnitudes of SA-12 (on Bar 1) were gradually increased with respect to each variation of prestressing forces. This trend matched the observation of SA-11 and SA-21. Meanwhile, the RMSD amplitudes of SA-22 were significant under the prestress force PS3 (i.e., 8.09%), which was about twice as compared to that under force PS2 (i.e., 4.31%). The RMSD values were subsequently reached 14.45% under the force PS4, which was around twice as large as that under the force PS3. The change in the RMSD index of SA-22 under the prestress forces PS3–PS4 could be caused by the internal concrete damage that was developed to form the surface crack. The RMSD index then achieved the maximum value (i.e., 36.64%) under test case PS5, which was consistent with the surface crack inspection (see Figure 15b). From the experimental results, it can be concluded that the sudden variations of the SA sensor’s impedance responses were induced by the concrete damage.

#### 4.4.2. Discussion on SA Sensor’s Performance in PSC Anchorage Zone

From the analyzed results shown in Figure 18, it was observed that the variation in the prestress forces in the PSC anchorage zone was successfully detected via the impedance features acquired from the SA sensors. The SA sensors located close to the bearing plate showed higher sensitivity to the change of the applied forces, thus suggesting that the SA sensors should be placed near to the bearing plate to enhance the sensitivity to the variation of the prestress forces. 

The analyzed results also pointed out that the distinctive changes in impedance signatures of the SA sensors revealed the occurrence of internal concrete damage in the anchorage zone. The damaged location (around SA-22) coincided with the determined one in the numerical stress analysis (Section 2) and numerical impedance analysis (Section 3). It can be suggested that the tensile damage location in the anchorage zone could be successfully predetermined via numerical investigation. Moreover, the numerical and experimental results evaluated the feasibility of the SA sensors for internal damage monitoring in the target PSC anchorage zone under the abrupt variation of prestressing forces.

The change in impedance features (e.g., RMSD metric) could be used to quantify damage severity in the concrete anchorage [45]. This study focused on investigating the feasibility of SA sensors and their optimal locations for damage monitoring in the anchorage. To quantitatively assess damage levels, firstly, stress analysis should be conducted by applying concrete damage models to determine damage scenarios in the concrete anchorage. Impedance signatures should then be analyzed corresponding to concrete damage scenarios. Lastly, the relationships between impedance features (i.e., RSMD index) and applied forces should be analyzed to identify damage severities. These findings demand future study on quantitative assessment of damaged anchorage zone. 

## 5. Concluding Remarks

In this study, the feasibility of smart aggregate (SA) sensors and their optimal locations for impedance-based damage monitoring in PSC anchorage zones were numerically and experimentally investigated. Firstly, the numerical stress analyses were performed on the PSC anchorage to determine potential damage locations induced by a series of prestressing forces. Secondly, the concept of a PZT-embedded SA sensor for impedance monitoring in concrete structures was briefly described. Thirdly, the numerical impedance analysis was performed to explore the sensitivities of the smart aggregates under stress field variations and artificial concrete damage. Finally, the experiment on a real-scale anchorage zone was performed in order to evaluate the smart aggregate practicality for damage monitoring. For a series of prestress forces, the measured impedance signals were quantified to comparatively evaluate the damage location in the anchorage. 

The following conclusions could be drawn based on the numerical and experimental investigations of the PSC anchorage zone. The impedance responses of the PZT-embedded SA sensor were sensitive to inner concrete damage induced by the tensile bursting stress in the PSC anchorage. The optimal localization of the SA sensors should be designed near the spiral at 0.28 H from the bearing plate. The SA sensor close to the bearing plate yielded higher impedance responses induced by stress variations, thus confirming that higher stress change leads to greater impedance responses of the SA sensors. The study proved the applicability of the SA technique for impedance feature-based damage monitoring. As further work, the locations and severities of inner concrete damage should be investigated by the implementation of concrete damage models. The applicability of the SA sensor should also be evaluated for monitoring compressive damage in the PSC anchorage zones. Moreover, optimization techniques (e.g., genetic algorithm) should be applied to optimize the placement of SA sensors using impedance features under various damage types in the anchorage zone. 

## Figures and Tables

**Table 1:** Material properties of anchorage zone components.

Parameters	Anchor Head & Wedge	Pure Concrete
Mass density, ρ (kg/m^3^)	7850	2400
Poisson’s ratio, ν	0.3	0.2
Young’s modulus, E (GPa)	200	27.7
Compressive strength, σ_c_ (MPa)	460	30
Tensile strength, σ_t_ (MPa)	460	3.0

**Table 2:** Material properties of PZT 5A.

Young’s Modulus, E (GPa)	Poisson’s Ratio, ν	Mass Density,ρ (kg/m^3^)	Dielectric Constant, ε^T^_33_ (F/m)	Coupling Constant, d_31_ (m/V)	Damping Loss Factor, η	Dielectric Loss Factor, δ
62.1	0.35	7750	1.53 × 10^−8^	−1.71 × 10^−10^	0.0125	0.015

**Table 3:** Simulation scenarios for impedance analyses in the anchorage zone.

Case	Intact	Applied Force	SA Sensor	Simulation
1	0PS	0.8PS	SA_11_, SA_21_, SA_31_	SA’s impedance responses based on internal stress distribution
2	0PS	1.2PS ^(^*^)^	SA_1j_, SA_2j_, SA_3j_	SA’s impedance responses based on tensile concrete damage

^(^*^)^ Artificial concrete damage was assigned at the vicinity of SA_21_.

**Table 4:** Design components of concrete anchorage.

Concrete Properties	Mixture 1 ^(^*^)^	Mixture 2 ^(^**^)^
Sand (kg)	80.1	81.8
Aggregate (D_max_ 25) (kg)	99.8	98.7
Cement (kg)	34.6	33.2
Water (liter)	15.6	16.1
Tested concrete slump (cm)	11.0	18.5
Tested compressive strength, σ_c_ (MPa)	23.3	16.7

(*) Mixture 1 (excluding coarse aggregate) was used for fabrication of SA sensors. (**) Mixture 2 was used for fabrication of concrete block of anchorage zone, see Section 4.2.

**Table 5:** Material properties of concrete anchorage, rebar, steel strand, adhesive, and PZT patch.

Properties	Concrete	Rebar	Steel Strand	Epoxy	PZT 5A
Young’s modulus (GPa)	20.7	200	197	0.74	62.1
Poisson’s ratio	0.20	0.33	0.33	0.38	0.35
Mass density (kg/m^3^)	2400	7850	7850	1090	7750
Compressive strength (MPa)	16.7	-	-	32.3	-
Yield strength (MPa)	-	400	1860	-	-

## Data Availability

Data available on reasonable request from the corresponding author.
